# Functional Dissociation of Ongoing Oscillatory Brain States

**DOI:** 10.1371/journal.pone.0038090

**Published:** 2012-05-30

**Authors:** Neda Salari, Christian Büchel, Michael Rose

**Affiliations:** Department of Systems Neuroscience, University Medical Center Hamburg Eppendorf, Hamburg, Germany; Centre national de la recherche scientifique, France

## Abstract

The state of a neural assembly preceding an incoming stimulus is assumed to modulate the processing of subsequently presented stimuli. The nature of this state can differ with respect to the frequency of ongoing oscillatory activity. Oscillatory brain activity of specific frequency range such as alpha (8–12 Hz) and gamma (above 30 Hz) band oscillations are hypothesized to play a functional role in cognitive processing. Therefore, a selective modulation of this prestimulus activity could clarify the functional role of these prestimulus fluctuations. For this purpose, we adopted a novel non-invasive brain-computer-interface (BCI) strategy to selectively increase alpha or gamma band activity in the occipital cortex combined with an adaptive presentation of visual stimuli within specific brain states. During training, oscillatory brain activity was estimated online and fed back to the participants to enable a deliberate modulation of alpha or gamma band oscillations. Results revealed that volunteers selectively increased alpha and gamma frequency oscillations with a high level of specificity regarding frequency range and localization. At testing, alpha or gamma band activity was classified online and at defined levels of activity, visual objects embedded in noise were presented instantly and had to be detected by the volunteer. In experiment I, the effect of two levels of prestimulus gamma band activity on visual processing was examined. During phases of increased gamma band activity significantly more visual objects were detected. In experiment II, the effect was compared against increased levels of alpha band activity. An improvement of visual processing was only observed for enhanced gamma band activity. Both experiments demonstrate the specific functional role of prestimulus gamma band oscillations for perceptual processing. We propose that the BCI method permits the selective modulation of oscillatory activity and the direct assessment of behavioral consequences to test for functional dissociations of different oscillatory brain states.

## Introduction

The state of a neural ensemble directly preceding an incoming stimulus has a prominent impact on the quality of the processing of that stimulus. A relevant amount of the variability of human task performance can be attributed to intrinsic fluctuations of neural activity prior to actual task processing [1], [2], [3], [4], [5], [6]. These intrinsic fluctuations can be characterized by the frequency range of ongoing electrophysiological activity as measured with EEG.

Prestimulus oscillations in variable frequency ranges have been shown to influence visual performance [4], [6], [7], [8], [9]. Studies have indicated that a high amount of low frequency oscillations, i.e. the alpha band impair perception [4], [10], [11] while gamma band frequencies (around 40 Hz) have been observed to enhance visual perception [12]. Nevertheless, other studies also indicate a functional relevance of alpha band oscillations for visual processing [13], [14], [15], [16].

These results suggest that neuronal oscillations with different frequencies within specific brain areas have a strong influence on the subsequent information processing.

However, the observation of prestimulus oscillatory activity in correlation to distinct information processing characteristics is not sufficient evidence to claim a causal relevance.

In particular for the gamma band oscillations it is currently discussed whether gamma band activity has an important functional role or can be regarded as a simple byproduct of neuronal processing [17].

To establish a more causal relation between ongoing oscillatory activity and information processing several methods have been used to directly modulate electrical brain activity and examine the consequences for subsequent stimulus processing. Exogenous activation of interneurons using optogenetic techniques in animals, for example, can induce gamma oscillations and was shown to affect sensory processing [18], [19]. Other studies have used direct electrical stimulation and shown that induced low frequency oscillations resulted in impaired visual detection [5] or improved memory consolidation during sleep [20]. Apart from these methods, several studies have shown that attention enhances oscillatory activity including gamma band activity during attended auditory [21] and visual stimuli [22], [23], [24]. However, the specificity of most of these methods is limited with respect to the time-, frequency- or space- domain, i.e. it is often not possible to estimate modulated oscillatory activity directly preceding stimulus presentation or to assess the expansion of the induced activity to other frequencies or locations (TMS or attentional modulations). However, a recent TMS study controlled for the specificity of frequency modulation by simultaneously recording EEG [25]. Classical neurofeedback approaches evoked modulations of oscillatory activity during training and tested the behavioral effects after a considerable time delay [15], [26].

Here we propose a novel non-invasive approach to examine the relevance of ongoing activity by an online estimation of the actual oscillatory brain state and an adaptive presentation of task stimuli within well characterized brain states. To enhance natural fluctuations of neural activity within well described brain states, participants were trained to voluntarily modulate different frequencies at distinct locations using a neurofeedback method based on continuously measured EEG. The identical brain-computer-interface (BCI) implementation used for neurofeedback allows the online calculation of the oscillatory activity and can adaptively present stimuli directly within the estimated oscillatory state without a considerable time delay. Using these methods, we focused on the role of gamma band oscillations over the lateral occipital cortex (LOC) for the subsequent processing of visual object stimuli. The LOC is known to be involved in the critical perceptual process of object detection [27], [28], [29] and this process can be modulated by top down processes [30]. Furthermore, the gamma band around 40 Hz is reported to play a functional role for object processing [12]. Therefore, we aimed to modulate oscillatory activity over the LOC to test for a functional relation. In particular the gamma band is discussed within several frameworks as an important parameter that can be used to directly assess neural information processing [31], [32]. The alpha band activity is also discussed to play an active role in visual processing [4], [8], [13], [14], [16]. Since previous models have indicated a different role of gamma and alpha band frequencies for perceptual and memory related information processing we used our method to examine common and dissociable effects of ongoing alpha and gamma band oscillations. Based on these models, we hypothesize that only increased prestimulus gamma band activity enhances the perceptual processing of subsequent visual stimuli, while increased prestimulus alpha band activity should not affect or even impair visual object processing.

In the first part of all experiments the neurofeedback approach was used to train volunteers to gain influence on the chosen frequency band within the LOC. The results show that the approach resulted in a modulation of oscillatory activity with a high degree of specificity regarding frequency and topography. In particular, the gamma band modulation was restricted to the targeted frequency range within the visual cortex. In the second part of the experiments the BCI was used to present task stimuli (noisy object images) with a high temporal resolution directly within modulated brain states, i.e., as increased gamma band activity is classified within the passed second of EEG measurement, the stimuli is immediately presented. The quality of the subsequent processing of the stimuli was assessed at the behavioral level using the detection rate of the objects within the noisy images and with a surprise recognition task after the object detection task.

The adaptive stimulation in direct temporal relation to the manipulation of the ongoing activity allows an estimation of the immediate consequences for behavior. The modulation of different brain states combined with the adaptive stimulation resulted in clearly dissociable effects for the subsequent information processing allowing a more direct functional characterization of ongoing oscillatory activity at different frequencies. The first experiment evaluated the feasibility, precision and behavioral consequences of BCI-evoked gamma band oscillations and the second experiment examined the specificity of the findings by comparing the effects between evoked gamma and alpha band oscillations.

## Results

### Experiment I

#### Selective modulation of ongoing gamma band activity over the visual cortex

In the first part of the experiment the neurofeedback approach was used to train volunteers to increase gamma band oscillations (30–45 Hz) over the lateral visual cortex in electrodes PO7/PO8 (by presenting the actual parameter value back on the monitor in front of the volunteer with a time delay of 1 s (see Materials and Methods section)). During training, the participant was rated as successful in increasing gamma band activity, if the continuously refreshed parameter value was above 0% and therefore above the level of the passive baseline. To evaluate the overall success of the gamma band training in each subject, the last training day was analyzed. In order to be included in further analysis the subject had to increase the parameter value in the majority of feedback segments (more than 60% of segments). Furthermore, an increase across training was assumed to reflect a successful training. With a training over three weeks (one hour/week) 12 of 20 participants showed a large increase in their ability to intentionally increase activity in the gamma band (percent feedback segments above baseline, successful group: last day: 84%±3.9 (s.e.m); unsuccessful group: last day: 34%±4.4 (s.e.m)). For the 12 successful participants the amount of segments with a mean gamma power higher than in the passive period increased with practice (repeated measures ANOVA, factor days, F(2,22) = 3.74, p = 0.04).

The EEG over the whole cortex was analyzed offline in more detail to estimate the training effect independent of possible artifacts like microsaccades, eye movements or muscle activity.

**Figure 1:**
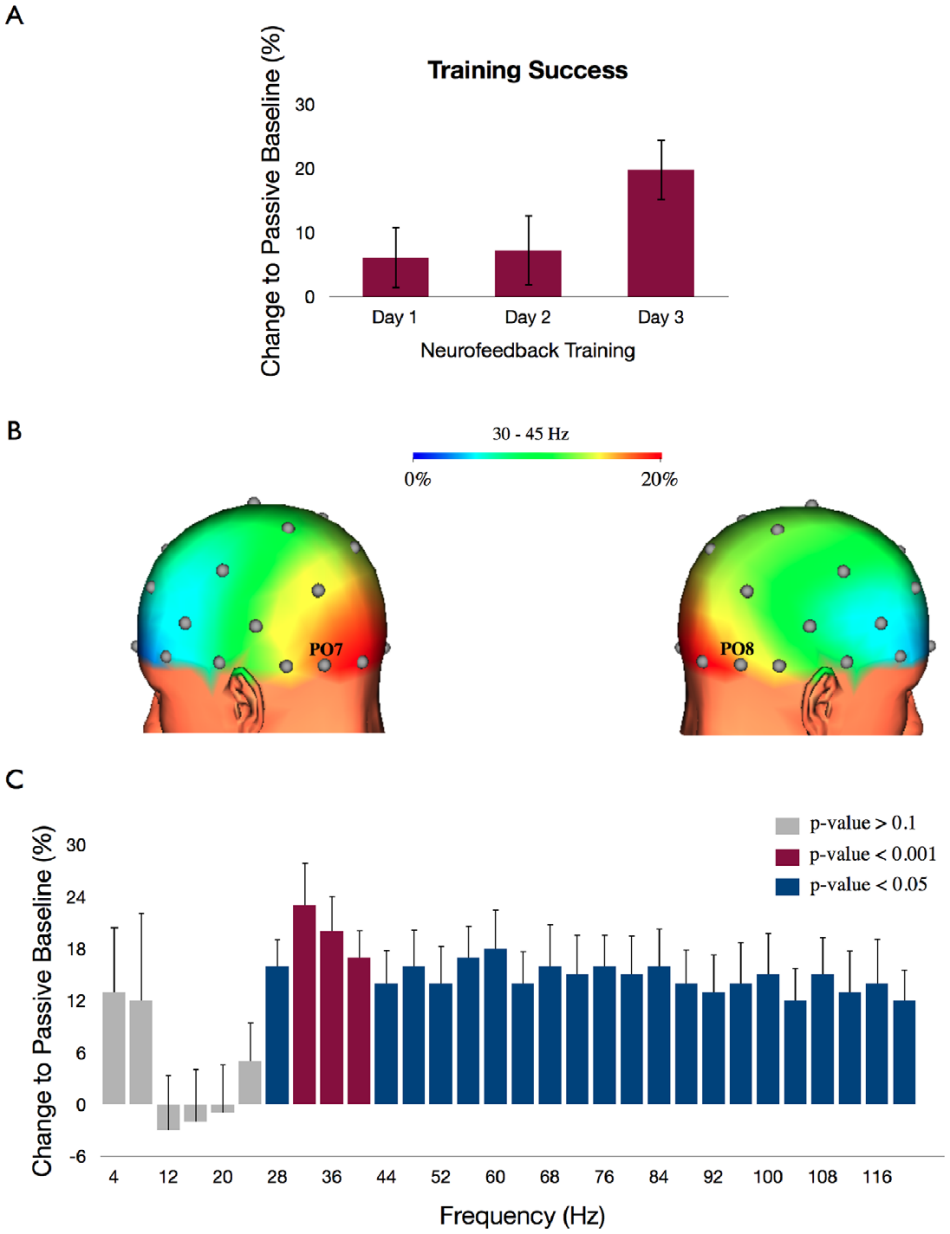
Topography and spectral specificity of evoked gamma-band oscillations. (a) Training success: Representation of the average change of gamma band activity (30–45 Hz) in electrodes PO7/PO8 during the training period compared to the passive period across training days. (b) Topographic representation of the average change of the gamma band within the last day. The maximum change is localized and limited at the electrodes over the occipital lobes that were used to calculate the training signal. (c) The percent change of power across frequencies from 0 to 124 Hz for the training periods compared to the passive periods within the last training session at the electrodes PO7/PO8. The different colors of the bars represent the significance of change to the passive baseline (t-tests for frequency bins of 4 Hz; Bonferroni corrected; gray bars: p-value>0.1, red bars: p-value<0.001, blue bars: p-value<0.05). The frequency distribution shows that the training effects were most prominent in the frequency range from 30–45 Hz (p<0.001) and extended to higher frequencies but no reliable changes were found in lower frequencies.

The offline analysis confirmed that the 12 volunteers were able to produce a reliably higher level of gamma band power in the last training session as compared to the passive period (t(11) = 3.09, p = 0.005). This effect increased with training as visualized in figure 1a (repeated measures ANOVA, factor days, F(2,22) = 12.22, p<0.001).

The recording of the additional EEG channels over the whole scalp allowed the calculation of the topographic specificity of the effect. This analysis revealed that the increase of gamma band power was limited to occipital electrodes and was not accompanied by a general increase of gamma band power over the whole scalp (repeated measures ANOVA, factors: condition (passive, training)×topography (pooled across central, frontal and parietal-occipital sites), F(2,22) = 5.47, p<0.05) (Fig. 1b). This topographic specificity is accompanied by a frequency distribution that shows that the gamma band training mainly influenced higher frequencies, most prominent in the trained frequency range 30–45 Hz (p<0.001). This effect extends to other high frequencies (p<0.05) however no differences were observed in lower frequencies (Fig. 1c). To assess the influence of gamma band training on the frequency spectrum we calculated the change of power in each frequency from 0 to 124 Hz (divided in bins of 4 Hz) for the feedback periods compared to the passive baseline within the last training session at the electrodes PO7/PO8. The significance of power change for each frequency bin to passive baseline was calculated by a t-test and corrected for multiple comparisons (Bonferroni, Fig. 1c). The tests revealed that the training effects were most prominent in the frequency range from 30–45 Hz (p<0.001) and extended to higher gamma frequencies but no reliable changes were found in lower frequencies (p<0.1). With an additional ANOVA analysis we were able to demonstrate the specific limitation of modulation in the trained gamma band frequencies (repeated measures ANOVA, factors: condition (passive, training)×frequency band (Alpha/Beta (8–20 Hz), Gamma (30–40 Hz), higher Gamma (>41 Hz)), F(2,22) = 4.68, p<0.05).

The results clearly demonstrate that this method can be used to selectively modulate neural activity in the gamma band over the visual cortex and did not affect oscillatory activity of other frequency bands below the gamma band.

A recent study has shown that scalp recorded gamma band oscillations in parietal electrodes in EEG-data can be influenced by microsaccades [33]. In order to assure the neural origin of the measured gamma band increase and to estimate the influence of saccadic activity, we applied a recently proposed saccadic spike potential (SP) detection method [34], which allows an accurate detection of microsaccades directly in EEG traces without acquiring fast eye-tracking (see Methods).

We exploited the saccade detection algorithm, to determine the amount and mean amplitude of detected SPs in both passive and feedback periods to test for saccadic changes between the periods and across training. In addition to the SP detection, a conservative method was applied to remove possible artifacts by eye movements or muscle activity (see Methods).

To ensure that microsaccades did not elicit the increase of gamma band activity in the training period, the amount and mean amplitude of microsaccades in both training and passive periods was compared. Within the last training session no changes were found between both periods regarding the SP amount (t(11) = 0.6, n.s.) or SP mean amplitude (t(11) = 1.18, n.s.). Furthermore, no changes were found across training (interaction across days, SP amount: F(2, 22) = 0.31, p = 0.73, SP amplitude: F(2, 22) = 0.21, p = 0.81), reassuring that the increased gamma band activity is not attributable to an increase of microsaccades but rather derives from neural activity.

#### Adaptive stimulation during different states of evoked ongoing gamma band activity

As a next step, we directly tested for the consequences of a deliberately increased gamma band power on subsequent information processing. Ten participants who successfully learned to control their gamma band activity participated in a slightly modified version of the initial training task. As in the first part of the experiment the parameter value of the gamma band activity was presented on the monitor in front of the volunteer to allow a deliberate influence of the gamma band within the visual cortex. We exploited the variability in evoking gamma band activity to test for a relation of prestimulus gamma band activity and visual object processing. Noisy images were adaptively presented in states of elevated or non-elevated ongoing gamma band oscillatory power instead of the parameter value (see Methods).

In each volunteer individual gamma band power levels were estimated from the parameter values achieved in the last training session (see Methods). When the value was higher than the upper gamma level (elevated gamma state) or lower than the lower gamma level (non-elevated gamma state) sometimes a phase scrambled image (33% visibility) was presented (see experimental design of experiment II). Thus, the subject was not informed about the actual success in this segment which was important to avoid a strategic bias on the image processing. The participant was instructed to detect an object within the scrambled version of the image by judging them as ‘living’ or ‘non-living’ by a button press. Participants were advised not to press a button if the object in the image was not detected. If ongoing gamma band activity over the visual cortex is causal for an enhanced processing of visual stimuli we expected that an increased level of prestimulus gamma band activity should improve the detection of noisy objects.

**Figure 2:**
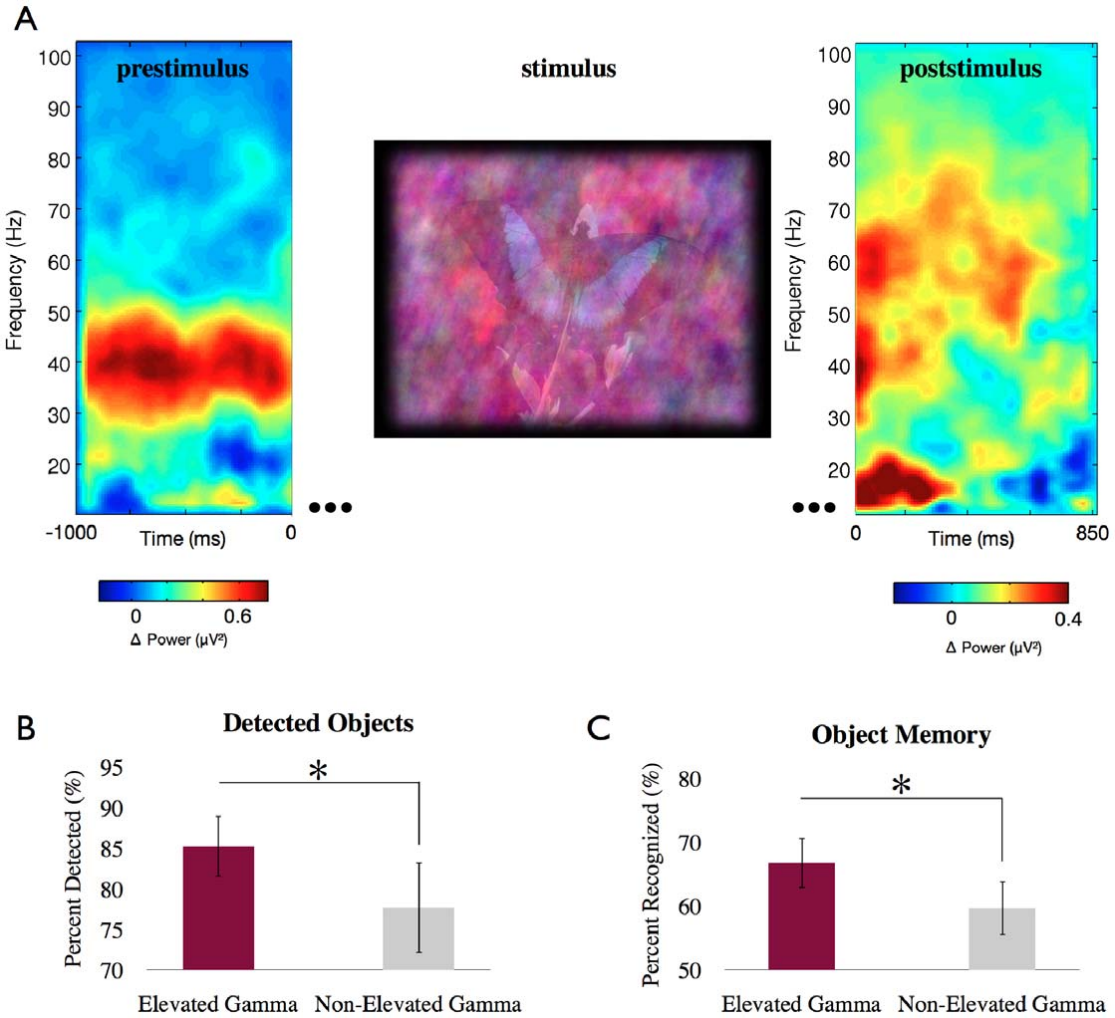
Time-frequency analysis of pre- and poststimulus periods and behavioral effects. (a) Time-frequency analysis of the total power difference between elevated gamma band and non-elevated gamma band states for electrodes PO7/PO8 before and after stimulus onset (grand mean over all participants). Significantly more gamma band activity in the exact trained frequency range (30–45 Hz) can be observed in the elevated gamma band state in the prestimulus period which resulted in significant more gamma band activity also after stimulus onset. The left and right time-frequency plot form a continuum, i.e. time point zero is identical for both plots. (b) Significantly more images were detected during elevated gamma states as during non-elevated gamma states. (c) The surprise memory task afterwards revealed significantly higher recognition rates for images detected during elevated gamma than non-elevated gamma states. (* p<0.05).

In accord with our assumptions the increased gamma band power resulted in a significant enhancement of visual object processing. During elevated gamma states more images were detected than during non-elevated gamma states (percent detected during non-elevated gamma band states: 77.7±5.6 (s.e.m) versus elevated: 85.23±3.8 (s.e.m); t(9) = 2.79, p = 0.02) (Fig. 2b). Participants were able to detect objects in the noisy images faster during elevated gamma band states than during non-elevated gamma band states. However, this effect did not reach a level of statistical significance.

The success of object processing was further examined in a subsequent surprise recognition task, performed 10 minutes after the experiment. In the recognition task participants unexpectedly had to judge images as ‘previously seen’ or ‘new’. Half of the images were already shown during the feedback session and the other half were not presented before. Hence, the recognition task was performed for all images of the detection task, regardless of whether they were detected or not. However, the analysis of the recognition task was based on images that were detected during the detection task and were correctly judged as ‘previously seen’ in the recognition task. Our results revealed significantly higher recognition rates for images that were detected during elevated gamma states than during non-elevated gamma states (percent correct non-elevated gamma state: 59.7±4.23 (s.e.m) versus elevated gamma state: 66.76±3.94 (s.e.m); t(9) = 2.69, p = 0.024) (Fig. 2c). For comparison the false positive rate (FPR) was assessed by calculating the percentage of ‘new’ images that were rated as ‘previously seen’. The FPR of 17% compared to 60% correctly remembered in the non-elevated gamma state and 67% in the elevated gamma state indicates reliable formation of memory for both states.

Both behavioral results clearly confirm that the increase of the gamma band power over the visual cortex enhanced the processing of the images.

The offline EEG analysis confirmed that the gamma band power was reliably different between the elevated and non-elevated gamma states (elevated gamma power against non-elevated gamma power, t(9) = 4.69, p<0.001). Thus, during the time period of 1 second before image presentation reliably more gamma band activity was evoked in the elevated gamma state compared to the non-elevated gamma state. A time-frequency analysis revealed that the increase of gamma band power during the elevated gamma condition was limited to the trained frequency range from 30–45 Hz and was not accompanied by increases in other higher or lower frequencies (Fig. 2a). We tested this statistically, using a nonparametric permutation test (Monte Carlo Method, 200 iterations) with a FDR correction for the time and frequency domain for electrodes PO7 and PO8. Results revealed that the gamma band increase was limited to the trained frequency range. The statistical tests were limited in the frequency (30–45 Hz) and time range of −300 ms to stimuli presentation.

This intentionally evoked gamma band increase during the elevated gamma band condition further resulted in an increased level of gamma band activity during the first 500 ms of subsequent image presentation (t(9) = 2.09, p = 0.03).

**Figure 3:**
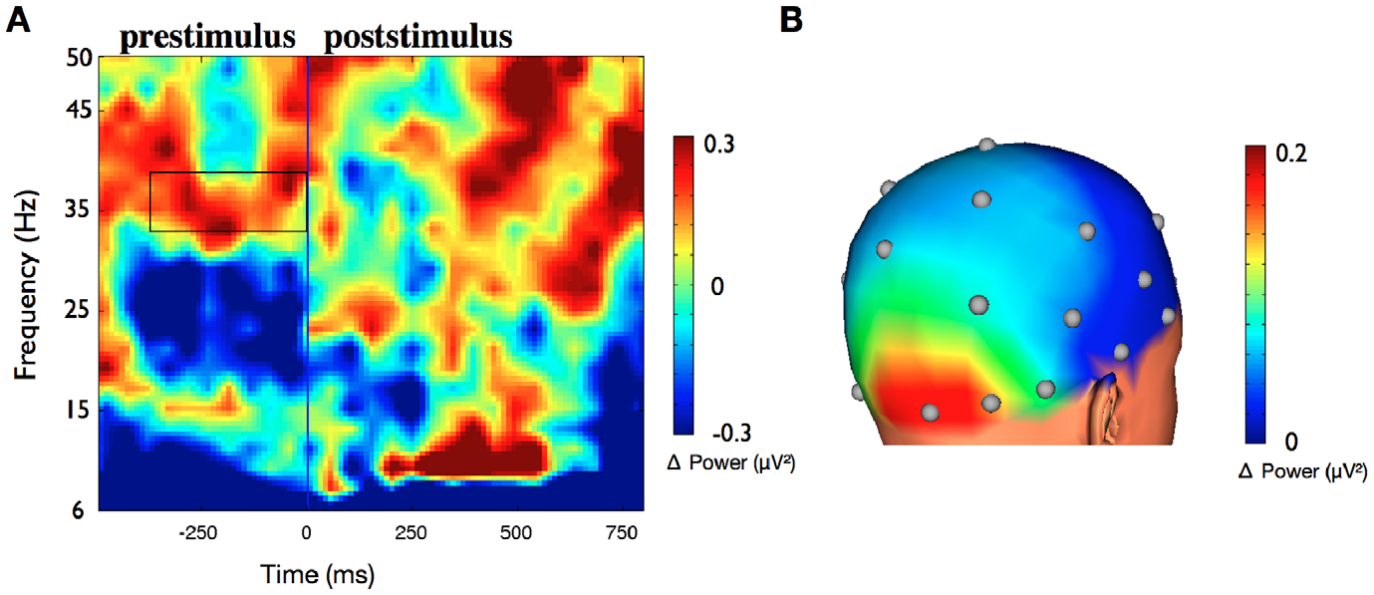
Time-frequency analysis and topography of hits compared to misses. (a) Time-frequency analysis of the difference between detected images (hits) compared to states of undetected images (misses) irrespective of the evoked oscillatory state (pooled over elevated and non-elevated gamma states) for electrodes PO7/PO8. The marked rectangle in the prestimulus period reveals a significant difference between both states (t(9) = 2.51, p = 0.03). No significant changes were found in the alpha (8–12 Hz) or beta band ranges (20–30 Hz). (b) Topographic representation of the prestimulus gamma band differences of detected images compared to undetected images. The maximum difference is localized in the occipital region.

A further time-frequency analysis compared detected images (hits) against undetected images (misses) independent from the evoked state (pooled over elevated and non-elevated gamma states) (Fig. 3a). The result showed a reliable power increase in the prestimulus period of the detected images limited to the gamma band (34–40 Hz, −400 – 0 ms, t(9) = 2.51, p = 0.03). No significant changes were found in the alpha (8–12 Hz) or beta band ranges (20–30 Hz). Hence, the different performance cannot be explained by additional prestimulus oscillatory activity of other frequencies. In addition, we performed a topography analysis of all electrodes for the prestimulus states of detected images compared to prestimulus states of undetected images. The analysis demonstrates that the difference between hit and miss states for the gamma band is topographically specific with a maximum difference in the occipital region (Fig. 3b). Thus, the prestimulus difference is restricted to the trained frequency range and brain region.

Importantly, results of the saccadic spike potential (SP) detection revealed no changes between the amount of detected SPs in the elevated and non-elevated gamma conditions one second before and after image presentation (F(1,9) = 0.33, n.s.). Analyses of the SP mean amplitude in both conditions again revealed no significant changes before and after image presentation (F(1,9) = 0.26, n.s.). Thus, results of the training as well as the object detection task demonstrated no influence of microsaccades for the deliberately evoked gamma band activity.

The comparison of the number and amplitude of saccades in the phases before and after stimulus presentation however revealed a significant increase in the amount of SPs (F(1, 9) = 9.39, p = 0.013) and SP amplitude (F(1, 9) = 21.92, p = 0.001) during stimulus presentation. Thus, our results confirmed the saccadic changes during stimulus presentation as reported previously [33] and therefore approve the sensitivity of the applied SP detection method.

### Experiment II

#### Selective modulation of ongoing gamma and alpha band activity in the visual cortex

Experiment II was designed to evaluate the specificity of the previous results for the gamma band oscillations. In experiment II volunteers were trained to deliberately switch between modulating gamma (30–45 Hz) and alpha (8–12 Hz) band oscillations in the visual cortex and noisy images were presented during increased alpha and gamma band activity as in experiment I. We aimed to disentangle the functional roles of the different frequencies and their influence on visual information processing.

**Figure 4:**
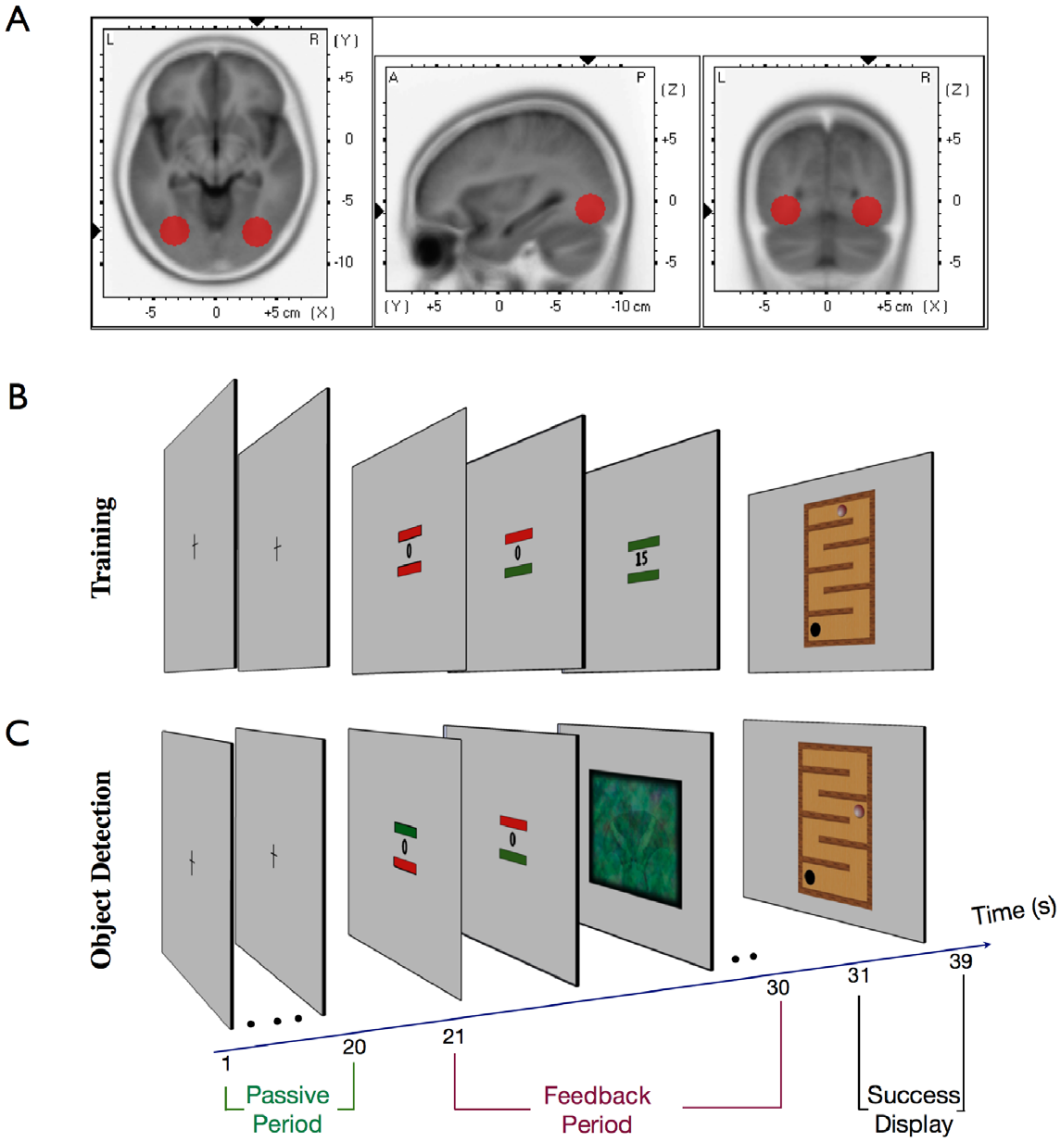
The region of interest and the experimental design. (a) Selected region of interest in the left and right LOC. (b) Training: During the passive period (20 seconds) volunteers fixated the central cross. This period was used to assess an actual baseline value of alpha or gamma current density power (CDP) in the ROIs. In the feedback period (second 21–30) participants tried to increase the CDP in the ROIs of bilateral LOCs (value at fixation) and at the same time avoid EOG (bar above value) and EMG (bar below value) artifacts by keeping the bars green. As an artifact occurred (at least one of the bars red) the presented value was set to zero. The success of the intentionally increased artifact-free gamma or alpha values was presented after the feedback period (success display). The ball moved along the track as a consequence of the successfully increased parameter values during the passed feedback period. The layout was identical for gamma and alpha band training. Both were trained in an alternating fashion with an information cue before the passive period that informed the volunteer about the relevant frequency band that should be increased. (c) Object detection: within periods of increased gamma or alpha band activity (depending on gamma or alpha session) instead of the parameter value sometimes a noisy version of an image was presented and the participant had to detect the object in the image.

As an attempt to further enhance the spatial precision of evoked oscillations the parameter value of this experiment was estimated using online source localization (LORETA, see Method) for a region of interest located in bilateral LOCs (sphere with a radius of 12 mm at left LOC: (x,y,z) = 34, −73, −8), right LOC: (x,y,z) = −34, −73, −8) (see Fig. 4a). Thus, every second the oscillatory activity of the relevant frequency (gamma or alpha) within bilateral LOC was estimated online and compared against baseline.

Furthermore, the design of the BCI was improved. Various artifacts (including microsaccades) were detected online and fed back to the volunteer by changing the color of the feedback bars around the parameter value (Fig. 4b). This should support the volunteer to develop an artifact-free strategy for the enhancement of neural activity for a more effective training.

**Figure 5:**
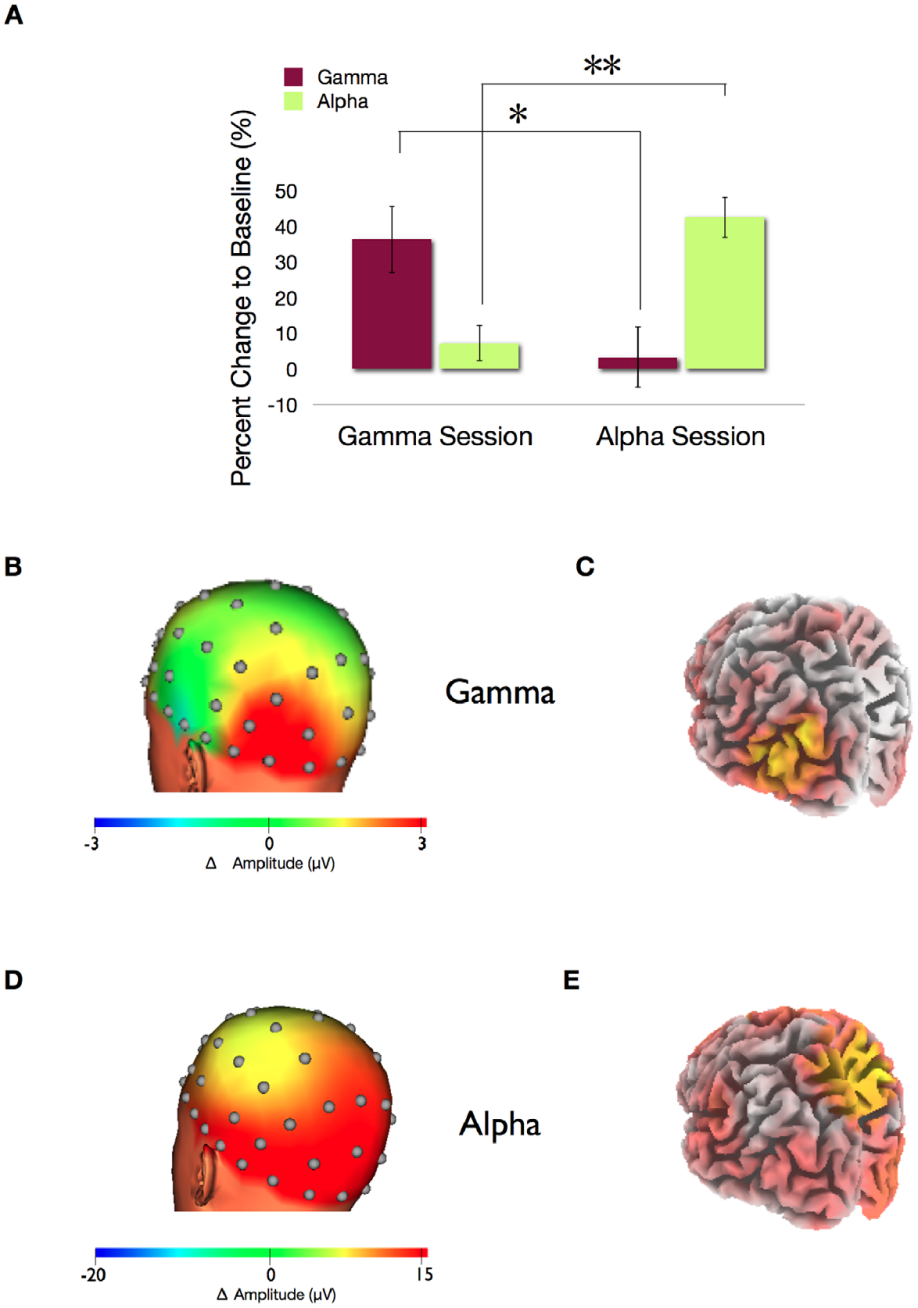
Topography and spectral specificity of evoked gamma or alpha band oscillations. (a) Percent change of gamma and alpha band activity in the selected ROI in the LOC in the gamma and alpha sessions within the last training day. Statistical tests revealed a higher increase of alpha power in the alpha sessions than in the gamma sessions (t(11) = 7.14, p<0.001) (** p<0.01) and a higher increase of gamma power in the gamma sessions than in the alpha sessions (t(11) = 2.75, p = 0.018) (* p<0.05). (b) On the sensor level: Topographic representation of the average change of the gamma band activity (30–45 Hz) during the feedback period compared to the passive period within the last training session. The maximum change is localized at the occipital lobes close to the trained ROIs. (c) Source estimation: sLORETA analysis of the subtracted amplitude of the gamma feedback sessions compared to the passive baseline. The yellow area represents the maximum estimated change of the gamma band activity to baseline. (Back view) (d) On the sensor level: Topographic representation of the average change of the alpha band activity (8–12 Hz) during the feedback period compared to the passive period within the last training session. The maximum change is localized at the occipital and parietal lobes. (e) Source estimation: sLORETA analysis of the subtracted amplitude of the alpha feedback sessions compared to the passive baseline. The yellow area represents the maximum estimated change of the alpha band activity to baseline. (Back view).

After three training sessions as in experiment I, offline analyses of the last training day showed that participants were able to selectively increase alpha and gamma band power in the defined ROI in the LOC. Analyses of the power change of gamma band activity to the passive baseline revealed a significant increase of gamma activity within the gamma sessions yet, not during the alpha sessions. Respectively alpha power change increased significantly during the alpha sessions however not during the gamma band sessions (interaction of session (alpha/gamma)×frequency band (alpha/gamma), F(1,11) = 33.75, p<0.001) (Fig. 5a). Volunteers were able to selectively increase gamma band oscillations in the gamma sessions as compared to the alpha band activity (t(11) = 3.4, p<0.01). No significant changes were found in the alpha band compared to baseline during gamma band sessions (t(11) = 1.41, n.s.). This selective increase was also reliable in the alpha band sessions as the alpha band was increased while the gamma band remained unaffected (t(11) = 4.33, p<0.01). The results show that volunteers learned to selectively influence each frequency band without affecting the other.

Results of the topographical and spatial distribution of the feedback effect revealed that the increase of alpha activity during the alpha sessions and gamma activity during the gamma sessions was increased at the occipital lobe (Fig. 5b,c). While increased gamma band activity in the gamma sessions was limited to the occipital lobe, results of the alpha sessions revealed a more widespread activation, as the alpha band increased in the occipital and parietal lobes (Fig. 5d,e).

#### Adaptive stimulation during different states of evoked ongoing gamma and alpha band activity

As in the first experiment, we directly tested for the consequences of selectively increasing gamma or alpha band activity on subsequent stimulus processing. Again, noisy object images (33% visibility) were adaptively presented in states of ongoing gamma or alpha band oscillation and volunteers should judge the detected objects as ‘living’ or ‘non-living’ by a button press (Fig. 4c). Individual alpha and gamma band activity levels were assessed from the last training that reflected the individual ability to increase the alpha and gamma band oscillations (see Methods). Thus, during training, the artifact-free (both bars green) parameter value was compared online against the determined individual level. When the value was higher than the level then sometimes a noisy image (n = 30 for alpha and for gamma band) was presented. Thus, images were only shown during artifact-free segments and a strategic bias on image processing was avoided as the participants were not informed about the actual success.

**Figure 6:**
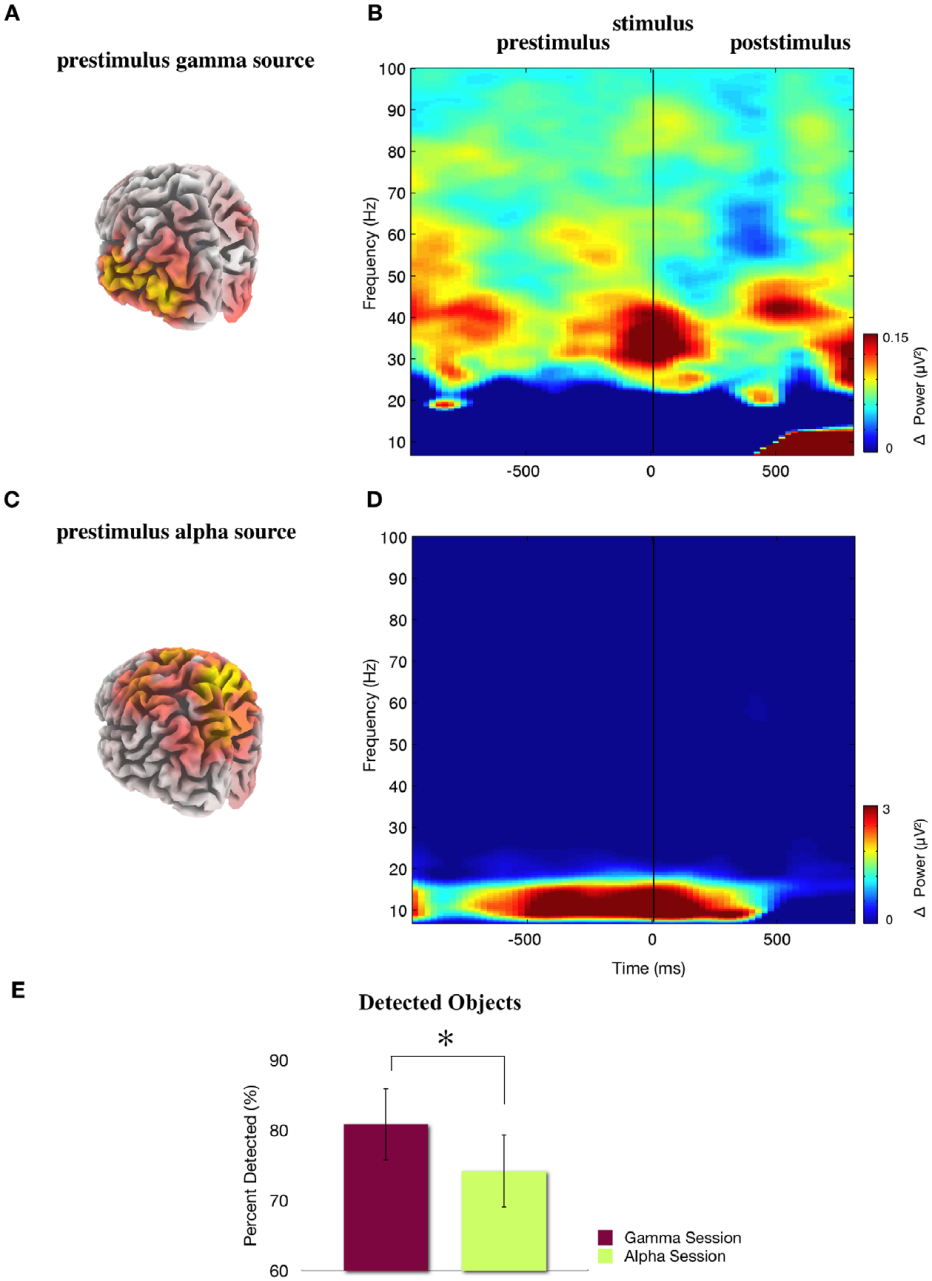
Analysis of pre- and poststimulus periods. (a) Source estimation: sLORETA analysis of the subtracted amplitude of the prestimulus gamma periods compared to the passive baseline. The yellow area represents the maximum estimated change of the gamma band activity to baseline. (Back view) (b) Time-frequency analysis of the total power difference between the gamma state compared to the alpha state for electrodes PO7/PO8 before and after stimulus onset (grand mean over all participants). A clear difference is found in the trained frequency range around 40 Hz. (c) Source estimation: sLORETA analysis of the subtracted amplitude of the prestimulus alpha periods compared to the passive baseline. The yellow area represents the maximum estimated change of the alpha band activity to baseline. (Back view) (d) Time-frequency analysis of the total power difference between the alpha state and the gamma state for electrodes PO7/PO8 before and after stimulus onset (grand mean over all participants). A clear difference is found in the trained frequency range around 8–12 Hz. (e) Significantly more images were detected during gamma band sessions than during alpha band sessions. (* p<0.05).

Our results clearly showed that participants were able to detect more images during increased gamma band oscillations than during increased alpha band values (percent detected during gamma sessions: 81±2.9 (s.e.m.) versus alpha band sessions: 74±3.6 (s.e.m.); t(11) = 3.03, p = 0.01) (Fig. 6e). As in experiment I, we applied the recognition task after BCI testing with 30 images shown during alpha session, 30 during gamma sessions and 60 additional new images. Analyses were identical to experiment I. However, no significant differences were found between images detected during alpha and gamma band sessions.

The offline EEG analysis confirmed that the prestimulus gamma and alpha band activity differed for alpha and gamma band sessions. The percent change of alpha and gamma activity to baseline was analyzed in both alpha and gamma band sessions in the trained LOC. Statistical tests revealed increased alpha power during the alpha sessions but not during the gamma sessions and increased gamma power during the gamma sessions but not during the alpha band related sessions (interaction of session (alpha/gamma)×frequency band (alpha/gamma) F(1,11) = 45.53, p<0.001). During gamma band sessions, gamma band was significantly increased as compared to baseline (t(11) = 3.1, p = 0.01), while alpha band activity remained unchanged (t(11) = 1.01, n.s.). A reverse effect was found during the alpha sessions as alpha band activity was significantly increased (t(11) = 4.26, p<0.01) and gamma band activity remained unaffected (t(11) = 0.46, n.s.) as compared to baseline. Thus, the modulation of both frequency bands remained highly selective as during the training part of the experiment.

Results of the topographical distribution of the neurofeedback effect for the prestimulus periods revealed similar results as the source data of the last training day. Increased gamma band activity was maximal and limited to the occipital lobe (Fig. 6a), while increased alpha band activity increased in occipital and parietal lobes (Fig. 6c).

In order to assure that the specific increase of gamma band activity in the LOC is accountable for the improvement of visual object processing, we calculated the change of alpha and gamma band activity during the prestimulus gamma band sessions also in the parietal region (x,y,z) = 15, −63, 65. This region is depicted from the results of source estimation, representing the maximum estimated change of the alpha band activity in the alpha sessions (Fig. 6c). However, during the prestimulus gamma band sessions we found no significant changes in the alpha or gamma band in the parietal region. Thus, the behavioral advantages of the gamma sessions cannot be explained by other oscillatory activity within parietal sites.

To further evaluate the effects of evoked oscillations within different frequency bands a time- frequency analysis was performed at the sensor level (as in experiment 1 for the electrodes PO7/PO8). A comparison of the oscillatory states directly preceding task stimulus presentation demonstrates the selective modulation of the different frequencies and the effects on the stimulus processing (Fig. 6b,d). The modulation of gamma band oscillations was clearly limited to the trained frequency range and did not affect lower frequencies and the alpha band oscillations were also restricted to the trained frequency range.

This was tested statistically, using a nonparametric permutation test (Monte Carlo Method, 200 iterations) with a FDR correction for the time and frequency domain in electrodes PO7 and PO8 for gamma states minus alpha states (Fig. 6b). The only significant differences between these two states in the prestimulus period were found in the exact trained frequency ranges of alpha (8–12 Hz) and gamma band (around 40 Hz).

## Discussion

The present study examined the functional relevance of gamma band oscillations in the visual cortex for subsequent visual object processing. The here established BCI approach offers a novel and non-invasive method for a selective manipulation of ongoing oscillatory activity and a direct examination of the consequences for neural processing and behavior by an adaptive stimulus presentation. The results demonstrate that different oscillatory brain states can be evoked by the approach with a high degree of specificity regarding time, space and frequency. The increase of gamma band oscillations improves the processing of a subsequent task reflected by behavioral and neural indicators. In the first experiment different levels of gamma band activity were compared and the results suggest a direct and important link between prestimulus gamma band activity and stimulus processing. The unique role of gamma band oscillations for visual processing is supported by the results of the second experiment. In a new group of volunteers we could replicate the relation of gamma band states to an improvement of processing in direct comparison to alpha band states.

Oscillations in the gamma band in the visual cortex have been linked to stimulus properties [32], [35], [36] and visual awareness [12], [37], [38]. However, the influence of these spontaneous fluctuations on visual performance remains controversial. Therefore, in the first experiment our approach was used to estimate gamma band oscillations and to reveal the effect of different levels of gamma band activity on cognitive performance. Within these different states of ongoing gamma band activity noisy images were presented as task stimuli. In accord with our assumptions, results confirmed that the increase of the trained gamma band activity over the visual cortex enhanced the processing of the visual objects. The behavioral advantage was observed for the detection of the objects in noise and for the recognition rate after the experiment. Both effects were related to an enhanced perceptual processing of stimuli presented at elevated levels of gamma band activity. The relevance of the prestimulus gamma band activity is further supported by the comparison of detected against missed images regardless of the actual state. The only reliable difference before the onset of the stimulus is located in the gamma band and in the occipital area. This is in accord with previous studies that have provided evidence that prestimulus gamma band activity in the visual cortex predict visual perception [8], [12], [38], [39]. Here, we tested this assumption by a direct manipulation of the prestimulus oscillatory activity and found clear evidence for a relevant influence of fast prestimulus oscillatory activity on subsequent task processing.

A recently proposed theory predicts that fast-spiking interneurons are responsible for the generation of gamma band oscillations resulting in rhythmic inhibition and a subsequent narrow window for effective excitation [18]. This indicates the important and functional role of gamma band oscillations for the establishment of a neural state within a circumscribed network that can facilitate the establishment of a new stimulus pattern within that network.

Studies in animals and humans have shown a top-down attentional influence on gamma band activity and a correlation to visual performance differences [23], [32], [40], [41]. However, a simple attentional mechanism cannot account for the specific increase of gamma band activity in the present study. This conclusion is supported by our results demonstrating the narrowness of the increased prestimulus gamma band activity (Fig. 2a) as a result of the frequency specific neurofeedback training, since attention has been shown to modulate gamma band oscillations in a broad frequency range from 30 up to 140 Hz [24]. Thus, the prestimulus frequency modulation was limited to the defined frequency range as a clear consequence of neurofeedback training.

The specific role of ongoing gamma band activity for visual processing is refined in the second experiment by the comparison to identical modulations within the alpha band. In contrast to the gamma band, prestimulus alpha activity is discussed to have an inhibitory role on perception [4], [8], [10]. In a recent study, occipital and parietal TMS at alpha frequency has been found to impair target detection in the visual field contralateral to the stimulated hemisphere [5]. Thus, in experiment II, noisy images were triggered and presented during real-time classification of increased alpha and gamma band activity in an alternating fashion. Although the absolute change was larger for the alpha band (Fig. 5d) the behavioral and neural indicators clearly showed an enhanced stimulus processing during gamma band states (Fig. 6e). Thus, in agreement with our assumptions, participant's detected more images during states of increased prestimulus gamma band activity as compared to states of increased prestimulus alpha band activity.

Results of the first experiment showed that the established elevated gamma band state not only improved processing of visual stimuli, but also improved memory recall of these visual stimuli. From all objects that were detected during the object detection task, participants recalled more objects that were presented during states of elevated gamma band activity than during non-elevated gamma band activity. A surprise recognition task was also performed in the second experiment, after participants had detected objects during increased gamma or increased alpha band states. However, no differences were observed between memory recall of images associated with increased alpha or gamma band activity. We assume that the comparable memory effect is related to different functional roles of alpha and gamma band activity.

Our results reveal convincing evidence that prestimulus gamma band oscillations improve perception. This is reflected in the amount of detected objects but also in a difference of memory performance between the two gamma band states. We suggest that the improvement in memory formation under the elevated gamma band state is also related to an enhanced perceptional processing that facilitates memory encoding. The results of the second experiment showed that alpha band activity did not improve perception and resulted in an equal memory formation for detected objects (the absolute amount of remembered objects is lower for alpha band states due to less detected objects during the object detection task). It could be assumed that the equal memory performance is related to the proposed functional role of alpha band oscillations and memory formation [14]. This assumption is supported by the results demonstrating that increased prestimulus alpha band oscillations remain increased in the poststimulus period and we suggest that memory encoding proceeds during the entire increased alpha state.

The equal memory performance in the second experiment thus could be related to an improved perceptual processing during elevated gamma band activity and to improved memorization of the objects during alpha band activity. Besides this more speculative interpretation, both experiments clearly demonstrate a functional relevance of prestimulus gamma band activity in the LOC for perceptual processing of visual objects.

Recent studies combining EEG and fMRI measurements support a relevant functional role of ongoing gamma band oscillations for visual processing. A positive correlation between gamma band oscillations and the fMRI response has been found in the visual cortex [42]. In contrast, alpha band oscillations have been shown to decrease the fMRI response in occipital areas [42], [43], [44], [45], [46]. These correlative findings suggest a relation of gamma band oscillations and neural activity in the LOC during the processing of visual object information [30]. This assumption is supported by our results demonstrating that an increase of ongoing gamma band oscillations in the LOC leads to improved visual object processing as compared to ongoing alpha band oscillations.

The results of both experiments show that gamma band oscillations could be selectively modulated by our BCI approach. In both experiments, the effect of neurofeedback training was maximal in the trained frequency range and prestimulus periods at testing revealed a narrow increase of gamma band activity limited to the trained frequency range. This demonstrates the potential of the BCI approach for a frequency specific modulation of ongoing oscillatory activity. Several methods including transcranial magnetic stimulation (TMS) [5], [20], [47], transcranial alternating current stimulation (tACS) [48] [47] [49] and attention [22], [23], [24] have been implied as methods to manipulate oscillations in different frequency bands. However, in most of these studies, the spectral effects do not correspond to the predefined frequencies, but also affect other frequencies. A recent TMS EEG study has assured a frequency specific phase modulation in the alpha band range before stimulus onset [25]. Apart from these methods, several studies have applied a feedback approach to train participants to increase various activity in different frequency ranges and tested cognitive performance effects [14], [15], [26]. In all of these studies performance tests were applied offline after neurofeedback training without an assessment of the actual oscillatory state preceding the actual behavioral test. Therefore, in our BCI method visual performance tests were carried out online during the estimation of alpha or gamma band oscillations. We assured the presentation of visual stimuli during modulation in the exact trained frequency range and topographic area.

In experiment II, the distribution of the estimated three-dimensional electrical activity of the gamma band increase to baseline showed a selective enhanced effect in the visual cortex as suggested by the topography in experiment I. Results of the topographical and spatial distribution of the alpha band increase demonstrated a rather widespread enhanced effect in the trained lateral occipital and in the occipito-parietal region, with a maximum effect in the superior parietal lobe. Previous studies used source based approaches to generate a feedback signal to enhance low beta and to suppress low alpha in the anterior cingulate cortex (ACC) [50]. Based on this study, a further study explored the effect of training in the ACC on anterior regions [51]. The present analyses showed that the selection of a region does not assure a maximal effect in the defined location. Therefore, post-hoc analyses are essential to evaluate other possible sources that may have affected the results. Our results are in agreement with previous studies implicating the origin of posterior alpha rhythm from occipito-parietal areas, where it is modulated by visual input [52], [53], [54]. In order to test whether visual object processing was specific to an increase of gamma band activity in the LOC, we tested whether results hold true for parietal regions. Results demonstrated that no significant changes were found in the gamma or alpha band range in the parietal regions during prestimulus gamma sessions. Therefore, alpha activation in parietal regions cannot explain the improvement of visual object processing.

In contrast to external stimulation or attentional manipulations, our BCI approach offers the opportunity to selectively modulate a predefined frequency range and examine behavioral and neural consequences by stimulation within well-described brain states that can be estimated and characterized in direct temporal relation to the stimulation. This online calculation allows the adaptive stimulation within predefined neural states.

The reported effects may be specific for perceptual processing, as studies have shown that functions like memory processing may be supported by oscillatory states of frequencies within the theta band [55], [56]. These assumptions can be tested using the developed BCI method, as the resulting high specificity regarding frequency range and location of gamma and alpha band training underlines the value of the BCI approach as a method for the examination of a more direct relationship between oscillatory brain states and behavior.

In summary, the actual study introduces a new method that combines neurofeedback training with an adaptive stimulation. The results demonstrate the value of this method for a highly selective modulation of gamma band activity that allows disentangling the functional relation of prestimulus gamma and alpha band activity for visual object processing. The results support a strong functional relation of prestimulus gamma band oscillations and the perceptual processing of visual object information within the LOC.

## Materials and Methods

### Ethics Statement

All experiments were approved by the ethics committee of the German Psychological Society (Deutsche Gesellschaft für Psychologie) and subjects gave written informed consent prior to the experiment.

### Experiment I

#### Subjects

Twenty healthy, right-handed volunteers with normal or corrected to normal vision participated in the experiment (mean age 32 years, range 20–40, 10 females). Ten successfully trained subjects participated in the second part of the experiment, the object detection task (mean age 32 years, range 20–40, 4 females). None of the participants had prior BCI experience.

#### Data collection

EEG was measured from 28 channels at standard locations (PO7, PO8, F3, F4, C3, C4, P3, P4, O1, O2, F7, F8, T7, T8, P7, P8, Fz, Cz, Pz, Fp1, Fp2, CP1, CP2, FC5, FC6, CP5, CP6, CPz) (BrainVision amplifier and software) and the signal from electrodes PO7 and PO8 were used for the calculation of the feedback signal. The EEG of the other electrodes was used for later offline analyses. All 28 channels were referenced to linked mastoids. In addition, we recorded vertical and horizontal EOG from above versus below the left eye (supraorbital VEOGS and infraorbital VEOGI) and from the outer canthi of the eyes (left HEOGL, right HEOGR), for detecting eye movements. Neck muscle activity was derived bipolarly about 20 cm below the occipital electrodes over the trapezius muscle. Electrode resistance was kept below 10 kOhm. EEG and EOG were amplified in the range from 0.03 Hz ( = 5 s time constant) to 124 Hz and A/D converted at 250 Hz sampling rate.

### Part I: Training Procedure

A task period consisted of a passive period (10 s) followed by a feedback period (10 s). Within both periods the analogous voltage change of 28 channels was A/D converted and the acquired EEG data was transmitted to the feedback PC via TCP/IP. A Fast Fourier Transform (FFT) was performed every second in electrodes PO7/PO8 and the summed gamma power for the frequency range 30–45 Hz was computed online.

#### Passive Period

In the passive period the participants screen displayed a central fixation cross. During the passive period participants fixated the central cross and were inactive. This period was used to assess an actual baseline value of gamma band power.

#### Feedback Period

During the feedback period the percentage change to the passive baseline was computed and this gamma change value (parameter value) was visually fed back to the participant at fixation (instead of the cross). The parameter value was recalculated every second so that the participant was constantly informed about the actual change of gamma band activity. Thus, in the feedback period the participants tried to increase the gamma band power by increasing the value at fixation. In order to avoid eye movements the parameter value was displayed at the fixation point during the feedback period. Participants were instructed to fixate the centre in both periods.

During the feedback period, the participant was successful in increasing gamma band activity, if the parameter value was above 0% for the majority of feedback segments (one segment = one second of feedback period).

#### Success Display

After each feedback period, the success of the feedback effort was presented as a bar graph indicating success (green) or failure (red) to increase the mean gamma band activity in the feedback period. The width of the bar represented the mean parameter value. Additionally, the success display served as a short break for the subjects between each task period (duration 6 seconds).

Four sessions with 11 task periods each, were presented with short breaks between the sessions resulting in a duration of about an hour for the whole experiment. Each subject performed the feedback training once a week over a period of 3 weeks.

### Part II: Object detection during different states of evoked ongoing gamma band activity

The experimental layout of the object detection task was identical to the training task procedure. The only difference was that scrambled images of 33% visibility were sometimes shown during the feedback period instead of the parameter value. The appearance of an image depended on the subject's performance in the ability to control the gamma band. The individual ability to control gamma band was assessed for each person by sorting the values of the last training session (percent change to passive period) and calculating the maximum of the lower third and the minimum of the upper third as indicators for non-elevated and elevated gamma levels. The actual value during the feedback period fluctuated depending on the participant's actual success in increasing the presented gamma value, which resulted either in elevated (successful) and non-elevated (less successful) gamma band activity. Thus, the value was compared online against the determined elevated and non-elevated gamma levels.

When the value was higher than the upper gamma level or lower than the lower gamma level a phase scrambled image (size: 10×7.5 cm, 33% visibility) was shown for 2 seconds instead of the feedback display.

#### Image selection

For the object detection task, 120 different images (size 336×252 pixels; 24-bit color depth) were selected from a database of natural scenes and a database with object images. Visibility of images was modulated by scrambling them according to a method described previously [30]. In short, each image was transformed into the amplitude and phase component by a Fourier transform for each RGB color channel and a fraction (here 33%) of the image phase was manipulated before transforming the amplitude and phase components back into image space.

Four sessions with 11 task periods each, were presented to assure the presentation of a maximum of 30 images for elevated gamma and 30 for non-elevated gamma. Overall, 60 images were shown during the object detection task and 60 additional images were used for the recognition memory task afterwards.

#### Recognition memory task

After the object detection task, a surprise recognition task was presented, which entailed the presentation of the 60 previously seen images with 60 new images. The images were randomized and presented in a pseudo randomized order while the participants had to judge images as ‘previously seen’ or ‘new’.

### Data Analysis

For the EEG offline analysis, data of all electrodes were segmented from each 10 s passive and feedback periods. To avoid effects evoked by the stimulus onset the beginning of the segment was set from 1000 ms after start of periods. Each passive and feedback segment was then divided into equal size segments of one second. EEGs were corrected for blinks and eye-movement artifacts by subtracting both EOG channels weighted by their transmission coefficient [57]. For each feedback segment and the appropriate passive segment a FFT (Hanning window) was calculated.

The time-frequency analysis (Fig. 2a) was calculated for the trained electrodes PO7/PO8 based on a starting period of 1200 ms before and 1000 ms after image presentation for images shown during elevated and non-elevated gamma states. These time courses were transformed using a time-frequency analysis based on multi-tapers using the fieldtrip toolbox (http://www.ru.nl/fcdonders/fieldtrip). This involved a sliding window with time steps of 50 ms and 2 Hz frequency bins from 10 to 100 Hz. Per frequency bin the window length was set at 256 ms and a spectral smoothing of 8 Hz, which resulted in three tapers per window.

#### Detection of microsaccades, EOG and muscle artifacts

We applied a SP detection method, which was previously tested using a combination of eye tracking and EEG to evaluate methods for attenuating its effects on EEG [34]. Ocular artifacts such as the SP are most prominent in the peri-orbital electrodes when referenced to occipital or parietal electrodes [58]. Thus, for the offline detection of SPs, a further “radial” electro-oculogram channel (REOG) was derived as recommended [34]. The REOG channel is defined as the average of all EOG channels referenced to Pz: REOG = (HEOGR+HEOGL+VEOGS+VEOGI)/4 – Pz

The REOG channel was filtered with a Butterworth IIR filter (BPF) of an order of 6, with a pass-band of 30–100 Hz. The detection threshold was set at 2 standard deviations from the mean of the filtered signal. The REOG trace yields reasonable accuracy for saccades above 0.2°, which should be sufficient to detect saccadic activity in visual paradigms [34].

To assure that an increase of absolute gamma power in the feedback period was not due to the influence of eye movements or neck muscle activity, the gamma band change in electrodes EOG and muscle was calculated and the maximum change was subtracted from the mean gamma power change in electrodes PO7/PO8 before any statistical test. Thus, a possible transfer of gamma band activity from EOG or muscle was eliminated.

### Experiment II

#### Subjects

12 healthy, right-handed volunteers with normal or corrected to normal vision participated in the experiment (mean age 25). None of the participants had prior BCI experience.

#### Data Collection

The volunteer sat in a separate room and watched a liquid crystal display monitor with a viewing distance of 1 m. EEG was measured from 58 active electrodes at standard locations (Fp1, Fp2, F3, F4, C3, C4, P3, P4, O1, O2, F7, F8, T7, T8, P7, P8, Fz, Cz, Pz, Oz, FC1, FC2, CP1, CP2, FC5, FC6, CP5, CP6, F1, F2, C1, C2, P1, P2, AF3, AF4, FC3, FC4, CP3, CP4, PO3, PO4, F5, F6, P5, P6, AF7, AF8, FT7, FT8, TP7, TP8, PO7, PO8, Fpz, AFz, CPz, POz) (ActiCap, Brain Products, Gilching, Germany) at sample rate of 250 Hz and all channels were referenced to Cz. VEOG, HEOG and neck muscle activity was derived as in experiment I.

#### Online Data Processing

In the second experiment we used a source based approach to train participants to selectively increase alpha and gamma band oscillations. Combining low-resolution electromagnetic tomography (LORETA) with the BCI technique has been demonstrated to derive more spatially specific information [50]. LORETA is an inverse solution technique which uses information acquired from electrodes placed on the scalp in order to estimate the distribution of electrical neural activity in three-dimensional space [59]. The region of interest was selected in the lateral occipital lobe (LOC), as gamma band oscillations in the LOC have been shown to facilitate visual processing [24]. In contrast, it was shown that alpha oscillations in occipital parietal areas impair visual processing [5].

Thus, the selected regions of interest were selected in the left LOC ((x,y,z) = 34, −73, −8) and right LOC ((x,y,z) = −34, −73, −8)) with a sphere of 12 mm, encompassing a total of 7.2 cm^3^ in each region of interest (ROI). The choice of the ROI was determined by previous EEG studies [30]. Thus, in experiment II the estimated current density power (CDP) in the LOC was used as the parameter value instead of the activity in electrodes PO7 and PO8 as in the first experiment.

The computation of the CDP in the LOC is calculated every 20 ms as we receive 5 data samples×58 channels with a sampling rate of 250 Hz. Since, the participants monitor was refreshed every second, the estimated (1000 ms/20 ms = 50) 50 CDP values within this second were added up and presented to the participant after 20 ms.

### Part I: Training Procedure

In experiment II, we conducted a slightly different experimental layout as in experiment I. Each session started with a passive period (20 s) followed by eight feedback periods (each 10 s).Thus, a mean passive baseline was calculated and used for the following eight feedback periods in a session.

The design of the experiment was similar to the first experiment with a cross during the passive period and a parameter value during the feedback periods. In the feedback period two bars were added above (eye artifacts) and below (muscle artifacts) the parameter value which informed participants on occurring artifacts within the past second. The bars were placed central and close to the parameter value, in order to keep the volunteer focused to the value and to avoid eye movement (Fig. 4b).

#### Passive Period

Therefore, during the passive period the participant fixated the central cross, while the mean gamma (during gamma sessions) or alpha (during alpha sessions) CDP in the defined ROIs was estimated and used as a relational index for the following feedback periods. Anytime a blink or eye movement occurred during the passive period, the corresponding segment (1 second) was removed to assure an artifact free baseline measurement period, If more than 20% of the passive period contained artifacts, then the session was stopped and a new session was started.

#### Feedback Period

During the feedback periods volunteers were instructed to increase the presented parameter value which expressed the percent change of gamma or alpha band (depending on gamma or alpha session) activity in the LOC. The parameter value was computed and refreshed with a time resolution of one second. We avoided a faster refresh of the value and color of the bars in order to avoid rapid perceptual changes that could manipulate neural activity. During the feedback period two bars monitored EOG (above bar) and EMG artifacts (below bar) occurring within the past second of feedback training (see section artifact detection). Thus, volunteers were informed about a successful increase of activity in the defined frequency range without an influence of artifacts if the value increased and the two bars turned green. Respectively, the bars turned red as EOG or EMG artifacts occurred and the parameter value was set to zero (Fig. 4b).

#### Success Display

In order to keep the volunteers motivated, a game track was presented as a success display instead of a bar, which was displayed for 9 seconds. The position of the ball changed as a consequence of the intentionally increased values during the passed feedback period without an influence of artifacts. Thus, only values that were successfully increased during artifact free segments were used for ball movement. High values resulted in large distance movements of the ball, whereas low values resulted in shorter distance movements after a less successful feedback period. Hence, the success display was integrated in the design to keep the volunteers engaged and motivated, as larger ball movements were accomplished after a successful feedback period.

Volunteers were ambitious to reach the goal as fast as possible. Additionally, the success display served as a short break for the participants between each feedback period. Volunteers had eight feedback periods to reach the target. If the volunteer did not accomplish the goal within the given feedback periods, then the session was stopped and a new session was started.

Participants were trained for an hour once a week over a period of 3 weeks. Each training day consisted of 8 sessions of gamma band training and 8 sessions of alpha band training, which were presented in an alternating sequence. Before each training session, volunteers were informed with an information cue about the upcoming gamma or alpha session.

### Artifact Detection

During training it is important to address non-cerebral sources of artifacts. Undesired artifacts can establish significant changes in the EEG, and, thus, can change or manipulate brain signals [60], [61]. Two groups of artifacts are known to determine a serious problem for BCI applications: electrical activity generated by muscle contraction in jaw, neck or shoulders (EMG) and activity generated by eye blinks, eye movements (EOG) or microsaccades. Three artifact detection modules were implemented that constantly monitored EOG, microsaccadic and EMG activity.

#### EOG

For the online detection of eye blinks, the bipolar derived channel VEOG = VEOGS-VEOGI was used. Thresholds for blink detection and eye movement detection were established in a pilot study with 20 participants. The average value of detected blinks across all volunteers was 140 mV with a standard deviation of 104 mV. Thus, the threshold was set at 50 mV to assure the detection of smaller blinks.

For the detection of eye movement, the bipolar derived HEOG = HOEGR-HEOGL channel was utilized. While eye blinks produce spikes, vertical, horizontal and round eye movements produce square shaped EOG [62]. Therefore, as a value exceeded the threshold value (average amplitude = 64 mV, standard deviation = 34 mV, threshold = 20 mV), we furthermore specified that all following data values within 40 ms had to greater than the threshold value.

#### Microsaccades

For the online detection of microsaccades the REOG channel (as described above) was calculated online. As the REOG signal was computed we applied an online ‘running’ standard deviation to avoid memory access [63]. Thus, the standard deviation is refreshed with each incoming data from the passive and feedback periods (the success display period was excluded as the period served as a short break). We exploited the saccade detection algorithm to determine the amount and mean amplitude of detected SP's in both passive and feedback periods to test for saccadic changes between the periods and across training.

During the success display period volunteers were informed about their average SPs per second and SP amplitude in the two passive periods and in the passed feedback period. Thus, participants are informed if they exceed the average SP amount or amplitude in the passed feedback period.

#### EMG

Most common sources of EMG are muscles, when closing, opening or clenching the jaw. These muscle contractions generate high gamma frequencies, which are measured close to the temporal locations (T7, T8). Moreover, muscle contraction in the neck can generate high frequencies as well. To control for possible EMG contamination, channels T7, T8, and the bipolar derived neck channel were subjected to sixth order Butterworth filtering in the bandpass 70–80 Hz. During the passive periods the average activity in the channels T7, T8 and the neck was calculated and set as a baseline for the following feedback periods. Thus, if the percentage change of 70–80 Hz activity in the EMG channels during the feedback period was higher than in the ROIs, then the bar below the display value turned red.

#### Efficiency of artifact control

To assess the effect of artifact control during neurofeedback training we determined the amount of artifact contamined alpha/gamma segments (at least one of the bars red) within the first and last training days. At the individual level, four volunteers were successful in avoiding artifacts from the first training day; success was defined as 35% or less of feedback segments affected by artifacts. All others showed a poor performance in artifact control within the first day. However, for these volunteers in particular, our results revealed a significant decrease of artifact contamined segments across training (reduction of 17% from first to last training day, F(1,7) = 6,02, p<0.05). Thus, our results demonstrate the efficiency of artifact control during neurofeedback training as volunteers learned to control artifacts across training.

### Part II: Adaptive stimulation during different states of evoked ongoing gamma and alpha band activity

As in experiment I part II, the volunteers participated in object detection after three days of training. As in experiment I scrambled images of 33% visibility were shown during the feedback period of both alpha and gamma band sessions. The selection of images was identical to experiment I.

However, in this experiment individual alpha and gamma band levels were estimated in each volunteer rather than elevated and non-elevated levels as in experiment I. The individual alpha and gamma band levels were assessed by deriving the median positive parameter value for the last alpha training session and the last gamma training session. If the determined value exceeded 20 then a maximum level value of 20 was set. 30 images were shown during the gamma band sessions and 30 images during the alpha band sessions. An image was presented for 2 seconds instead of the feedback display. Participants identified an object within the scrambled image by a button press.

### Data Analysis

For the EEG offline analysis, data of all channels was first divided into passive and feedback periods. The first 1000 ms of both periods were removed in order to avoid effects evoked by stimulus onset. Each passive and feedback period was divided into equal size segments of one second. The data was preprocessed and controlled for artifacts as described for the online processing of data.

To evaluate the outcome of alpha and gamma band training with artifact control in the predefined ROIs within the last training day, we applied a LORETA transformation on the alpha and gamma band filtered channels. Artifact-free segments (EOG and EMG bars green) were extracted and the median (less sensitive to extreme distributed data values) percent change of gamma/alpha activity in the ROIs compared to baseline was derived. These segments were also controlled offline for artifacts that were possibly not detected. We conducted a repeated measures analysis of variance (ANOVA with factors session and frequency band) to compare the gamma and alpha band activity change during the alpha and gamma feedback periods.

To calculate the topographical distribution of BCI training, the electric potential differences (time domain EEG) in each electrode between the feedback and the passive period was calculated for both gamma and alpha periods within the last training day (subtracted amplitude). To estimate the three-dimensional distribution of electrical activity (current density) of gamma and alpha BCI training we applied sLORETA (The KEY Institute for Brain-Mind Research, Zurich: [64]) to the subtracted electric potential differences. The standardized LORETA method is more exact and achieves less localization error.

The time-frequency analysis (Fig. 6bd) was calculated for the channels PO7/PO8, as they are closest to the trained ROI. The analysis was calculated based on a starting period of 1200 ms before and 1000 ms after image presentation for images shown during alpha and gamma band states. As in experiment I, these time courses were transformed using a time-frequency analysis based on multi-tapers using the fieldtrip toolbox (http://www.ru.nl/fcdonders/fieldtrip). This involved a sliding window with time steps of 50 ms and 1 Hz frequency bins from 2 to 100 Hz. Per frequency bin the window length was set at 256 ms and a spectral smoothing of 8 Hz, which resulted in three tapers per window.

## References

[1] Hesselmann G, Kell CA, Eger E, Kleinschmidt A (2008). Spontaneous local variations in ongoing neural activity bias perceptual decisions.. Proc Natl Acad Sci U S A.

[2] Hesselmann G, Kell CA, Kleinschmidt A (2008). Ongoing activity fluctuations in hMT+ bias the perception of coherent visual motion.. J Neurosci.

[3] Fox MD, Snyder AZ, Vincent JL, Raichle ME (2007). Intrinsic fluctuations within cortical systems account for intertrial variability in human behavior.. Neuron.

[4] van Dijk H, Schoffelen JM, Oostenveld R, Jensen O (2008). Prestimulus oscillatory activity in the alpha band predicts visual discrimination ability.. J Neurosci.

[5] Romei V, Gross J, Thut G (2010). On the role of prestimulus alpha rhythms over occipito-parietal areas in visual input regulation: correlation or causation?. J Neurosci.

[6] Linkenkaer-Hansen K, Nikulin VV, Palva S, Ilmoniemi RJ, Palva JM (2004). Prestimulus oscillations enhance psychophysical performance in humans.. J Neurosci.

[7] Super H, van der Togt C, Spekreijse H, Lamme VA (2003). Internal state of monkey primary visual cortex (V1) predicts figure-ground perception.. J Neurosci.

[8] Hanslmayr S, Aslan A, Staudigl T, Klimesch W, Herrmann CS (2007). Prestimulus oscillations predict visual perception performance between and within subjects.. Neuroimage.

[9] Monto S, Palva S, Voipio J, Palva JM (2008). Very slow EEG fluctuations predict the dynamics of stimulus detection and oscillation amplitudes in humans.. J Neurosci.

[10] Ergenoglu T, Demiralp T, Bayraktaroglu Z, Ergen M, Beydagi H (2004). Alpha rhythm of the EEG modulates visual detection performance in humans.. Brain Res Cogn Brain Res.

[11] Busch NA, Dubois J, VanRullen R (2009). The phase of ongoing EEG oscillations predicts visual perception.. J Neurosci.

[12] Wyart V, Tallon-Baudry C (2009). How ongoing fluctuations in human visual cortex predict perceptual awareness: baseline shift versus decision bias.. J Neurosci.

[13] Babiloni C, Vecchio F, Bultrini A, Luca Romani G, Rossini PM (2006). Pre- and poststimulus alpha rhythms are related to conscious visual perception: a high-resolution EEG study.. Cereb Cortex.

[14] Klimesch W, Sauseng P, Gerloff C (2003). Enhancing cognitive performance with repetitive transcranial magnetic stimulation at human individual alpha frequency.. Eur J Neurosci.

[15] Hanslmayr S, Sauseng P, Doppelmayr M, Schabus M, Klimesch W (2005). Increasing individual upper alpha power by neurofeedback improves cognitive performance in human subjects.. Appl Psychophysiol Biofeedback.

[16] Lisman J (2005). The theta/gamma discrete phase code occuring during the hippocampal phase precession may be a more general brain coding scheme.. Hippocampus.

[17] Jia X, Kohn A (2011). Gamma rhythms in the brain.. PLoS Biol.

[18] Cardin JA, Carlen M, Meletis K, Knoblich U, Zhang F (2009). Driving fast-spiking cells induces gamma rhythm and controls sensory responses.. Nature.

[19] Sohal VS, Zhang F, Yizhar O, Deisseroth K (2009). Parvalbumin neurons and gamma rhythms enhance cortical circuit performance.. Nature.

[20] Marshall L, Helgadottir H, Molle M, Born J (2006). Boosting slow oscillations during sleep potentiates memory.. Nature.

[21] Tiitinen H, Sinkkonen J, Reinikainen K, Alho K, Lavikainen J (1993). Selective attention enhances the auditory 40-Hz transient response in humans.. Nature.

[22] Gruber T, Muller MM, Keil A, Elbert T (1999). Selective visual-spatial attention alters induced gamma band responses in the human EEG.. Clin Neurophysiol.

[23] Fries P, Reynolds JH, Rorie AE, Desimone R (2001). Modulation of oscillatory neuronal synchronization by selective visual attention.. Science.

[24] Tallon-Baudry C, Bertrand O, Henaff MA, Isnard J, Fischer C (2005). Attention modulates gamma-band oscillations differently in the human lateral occipital cortex and fusiform gyrus.. Cereb Cortex.

[25] Dugue L, Marque P, VanRullen R (2011). The phase of ongoing oscillations mediates the causal relation between brain excitation and visual perception.. J Neurosci.

[26] Keizer AW, Verment RS, Hommel B (2010). Enhancing cognitive control through neurofeedback: a role of gamma-band activity in managing episodic retrieval.. Neuroimage.

[27] Malach R, Reppas JB, Benson RR, Kwong KK, Jiang H (1995). Object-related activity revealed by functional magnetic resonance imaging in human occipital cortex.. Proc Natl Acad Sci U S A.

[28] Kourtzi Z, Kanwisher N (2001). Representation of perceived object shape by the human lateral occipital complex.. Science.

[29] Grill-Spector K, Kushnir T, Hendler T, Edelman S, Itzchak Y (1998). A sequence of object-processing stages revealed by fMRI in the human occipital lobe.. Hum Brain Mapp.

[30] Rose M, Schmid C, Winzen A, Sommer T, Buchel C (2005). The functional and temporal characteristics of top-down modulation in visual selection.. Cereb Cortex.

[31] Ray S, Maunsell JH (2011). Different origins of gamma rhythm and high-gamma activity in macaque visual cortex.. PLoS Biol.

[32] Engel AK, Fries P, Singer W (2001). Dynamic predictions: oscillations and synchrony in top-down processing.. Nat Rev Neurosci.

[33] Yuval-Greenberg S, Tomer O, Keren AS, Nelken I, Deouell LY (2008). Transient induced gamma-band response in EEG as a manifestation of miniature saccades.. Neuron.

[34] Keren AS, Yuval-Greenberg S, Deouell LY (2009). Saccadic spike potentials in gamma-band EEG: characterization, detection and suppression.. Neuroimage.

[35] Gray CM, Konig P, Engel AK, Singer W (1989). Oscillatory responses in cat visual cortex exhibit inter-columnar synchronization which reflects global stimulus properties..

[36] Siegel M, Konig P (2003). A functional gamma-band defined by stimulus-dependent synchronization in area 18 of awake behaving cats.. J Neurosci.

[37] Rodriguez E, George N, Lachaux JP, Martinerie J, Renault B (1999). Perception's shadow: long-distance synchronization of human brain activity.. Nature.

[38] Melloni L, Molina C, Pena M, Torres D, Singer W (2007). Synchronization of neural activity across cortical areas correlates with conscious perception.. J Neurosci.

[39] Womelsdorf T, Fries P, Mitra PP, Desimone R (2006). Gamma-band synchronization in visual cortex predicts speed of change detection.. Nature.

[40] Bichot NP, Rossi AF, Desimone R (2005). Parallel and serial neural mechanisms for visual search in macaque area V4.. Science.

[41] Steinmetz PN, Roy A, Fitzgerald PJ, Hsiao SS, Johnson KO (2000). Attention modulates synchronized neuronal firing in primate somatosensory cortex.. Nature.

[42] Scheeringa R, Fries P, Petersson KM, Oostenveld R, Grothe I (2011). Neuronal dynamics underlying high- and low-frequency EEG oscillations contribute independently to the human BOLD signal.. Neuron.

[43] Becker R, Reinacher M, Freyer F, Villringer A, Ritter P (2011). How ongoing neuronal oscillations account for evoked fMRI variability.. J Neurosci.

[44] Goldman RI, Stern JM, Engel J, Cohen MS (2002). Simultaneous EEG and fMRI of the alpha rhythm.. Neuroreport.

[45] Moosmann M, Ritter P, Krastel I, Brink A, Thees S (2003). Correlates of alpha rhythm in functional magnetic resonance imaging and near infrared spectroscopy.. Neuroimage.

[46] de Munck JC, Goncalves SI, Huijboom L, Kuijer JP, Pouwels PJ (2007). The hemodynamic response of the alpha rhythm: an EEG/fMRI study.. Neuroimage.

[47] Kanai R, Chaieb L, Antal A, Walsh V, Paulus W (2008). Frequency-dependent electrical stimulation of the visual cortex.. Curr Biol.

[48] Zaehle T, Rach S, Herrmann CS (2010). Transcranial alternating current stimulation enhances individual alpha activity in human EEG.. PLoS One.

[49] Pogosyan A, Gaynor LD, Eusebio A, Brown P (2009). Boosting cortical activity at Beta-band frequencies slows movement in humans.. Curr Biol.

[50] Congedo M, Lubar JF, Joffe D (2004). Low-resolution electromagnetic tomography neurofeedback.. IEEE Trans Neural Syst Rehabil Eng.

[51] Cannon R, Lubar J, Congedo M, Thornton K, Towler K (2007). The effects of neurofeedback training in the cognitive division of the anterior cingulate gyrus.. Int J Neurosci.

[52] Berger H (1929). Über das elektrenkephalogramm des menschen.. Arch Psycchiatr Nervenkr.

[53] Adrian ED, Matthews BH (1934). The interpretation of potential waves in the cortex.. J Physiol.

[54] Hari R, Salmelin R, Makela JP, Salenius S, Helle M (1997). Magnetoencephalographic cortical rhythms.. Int J Psychophysiol.

[55] Guderian S, Schott BH, Richardson-Klavehn A, Duzel E (2009). Medial temporal theta state before an event predicts episodic encoding success in humans.. Proc Natl Acad Sci U S A.

[56] Addante RJ, Watrous AJ, Yonelinas AP, Ekstrom AD, Ranganath C (2011). Prestimulus theta activity predicts correct source memory retrieval.. Proc Natl Acad Sci U S A.

[57] Gratton G, Coles MG, Donchin E (1983). A new method for off-line removal of ocular artifact.. Electroencephalogr Clin Neurophysiol.

[58] Boylan C, Doig HR (1989). Effect of saccade size on presaccadic spike potential amplitude.. Invest Ophthalmol Vis Sci.

[59] Pascual-Marqui RD, Michel CM, Lehmann D (1994). Low resolution electromagnetic tomography: a new method for localizing electrical activity in the brain.. Int J Psychophysiol.

[60] Ebrahimi T, Vesin JM, Garcia G (2003). Brain-computer interface in multimedia communication..

[61] Fatourechi M, Bashashati A, Ward RK, Birch GE (2007). EMG and EOG artifacts in brain computer interface systems: A survey.. Clin Neurophysiol.

[62] Krishnaveni V, Jayaraman S, Anitha L, Ramadoss K (2006). Removal of ocular artifacts from EEG using adaptive thresholding of wavelet coefficients.. J Neural Eng.

[63] Welford BP (1962). Note on a method for calculating corrected sums of squares and products.. Technometrics.

[64] Pascual-Marqui RD (2002). Standardized low-resolution brain electromagnetic tomography (sLORETA): technical details..

